# Physical demands and movement characteristics of veterans football players

**DOI:** 10.3389/fspor.2025.1602127

**Published:** 2025-09-04

**Authors:** Florian Egger, Vivian Graf, Shaan Kotecha, Sohag Saleh, Tim Meyer

**Affiliations:** ^1^ Institute of Sports and Preventive Medicine, Saarland University, Saarbrücken, Germany; ^2^ Imperial College School of Medicine, London, United Kingdom

**Keywords:** veterans football, cardiocirculatory strain, heart rate zones, field positions, movement patterns, step count, exercise intensity, video analysis

## Abstract

**Background:**

This study aimed to investigate physical demands and movement characteristics of veterans football (VF) players. VF players were recruited from the West and South London Leagues.

**Methods:**

A 15 m shuttle run test was used to assess maximum heart rate (HR_max_), and continuous heart rate was monitored to evaluate the cardiocirculatory strain during matches. Video analysis was performed to analyze movement characteristics, such as step counts, number of passes and sprints, changes of directions (COD), and standing time.

**Results:**

A total of 91 male VF players (age, 45 ± 6 years; BMI, 26.3 ± 4.0 kg/m^2^) participated in the study. The mean heart rate was 147 ± 14 min^−1^ corresponding to 80 ± 8% of HR_max_, with 57 ± 14% of match time completed above 80% HR_max_. Midfielders completed more sprints (90 ± 10) compared with forwards (34 ± 6, *p* < 0.001) and defenders (50 ± 10, *p* < 0.01). Standing time was significantly higher (*p* < 0.001) in forwards (740 ± 87 s) and defenders (649 ± 111 s) than in midfielders (181 ± 17 s). During a match time of 86 ± 36 min, players covered 5,790 ± 963 steps, equivalent to approximately 6 km, and made 120 ± 59 COD and 128 ± 62 passes.

**Discussion:**

The cardiocirculatory strain in VF football seems to be considerably high. Therefore, one VF match appears to be sufficient to meet the minimum of current guidelines on health-promoting activities. Position-specific differences in VF are evident for midfielders, who are potentially exposed to higher physical demands compared with other field positions.

## 1 Introduction

Veterans football (VF) provides an opportunity for passionate football players to continue harvesting the benefits of regular exercise, competition, and social interaction (1). Football *per se* provides significant cardiovascular benefits and reduces adverse cardiovascular risk factors (1–3). Positive health effects of regular football training include a reduction in body fat, peripheral vascular resistance, and, thus, blood pressure (2, 4, 5). In addition, football has been associated with improvements in microvascular endothelial function, muscle function, and postural balance (3–5). Nevertheless, the high-intensity interval character typical for football may even lead older individuals to exercise above 80% of their maximum heart rate (HR_max_) (3, 6, 7). However, it should be noted that repeated bouts of intense exercise potentially impose a risk for cardiovascular events with increasing age (8, 9).

While previous football studies examined mostly smaller cohort sizes of elderly players (3, 7, 10), information on the physical demands across a larger group of middle-aged players is scarce. Moreover, movement characteristics and positional differences of VF players remain unexplored. Therefore, this study aimed to examine the cardiocirculatory strain and video-based VF-specific movement patterns such as step counts (SC), number of passes and sprints, changes of directions (COD), and standing time in middle-aged players during a competitive match.

## 2 Methods

This cross-sectional, observational study was conducted between February 2022 and March 2023 in London, United Kingdom, in accordance with the Declaration of Helsinki and approved by the Imperial College Research and Ethics Committee and the Research Governance and Integrity Team (reference number 21IC7314).

### 2.1 Participants

The study population consisted of male VF players aged >35 years who regularly participated in league matches and training sessions. Female players were not included as female teams were not available in this region. Players were recruited from 11 teams from two leagues, namely, the West London Veterans Football League and Southern Veterans Football League. Each player provided written informed consent to participate in the study with the option to withdraw at any given stage. Goalkeepers were excluded due to their different activity profile compared with field players (11). Further exclusion criteria were injuries incompatible with play, acute infections, and severe internal diseases. Field positions were determined via individual interviews and verified 100% through video analysis of the matches.

### 2.2 Protocol

Data collection consisted of three steps for each player: (I) completion of completion of 15 m shuttle run measurements and an anthropometric questionnaire to determine age, height, weight, years of competitive football experience, and average weekly exercise hours, (II) measurement of resting heart rate before and HR_max_ during a 15 m shuttle run test prior to a training session (1 week before match), (III) continuous heart rate (HR) monitoring and video recording of VF-specific movement characteristics (SC, COD >90°, standing time, number of passes, and number of sprints) during one league match. A total of 12 matches were analyzed.

All matches were played on full-sized pitches, measuring 105 m by 68 m. Of the 12 matches, 9 were played on grass and 3 on artificial turf. Subjects were required to play football for at least 30 min during the monitored league match to be included in our analyses. All matches were organized by the West and Southern Veterans Football Leagues and officiated by a referee.

HR monitoring was conducted using HR chest-strap monitors (Wahoo TICKR Gen 2, Atlanta, GA, USA). The correct fitting of the chest strap connected with an HR monitor was always checked for each subject before the shuttle run test, sessions, and matches. HR was recorded continuously throughout the match, excluding halftime. Resting HR was recorded just before the training session by asking the participants to stand still for 5 min. This was followed by a 15 m shuttle run to determine HR_max_ and football-specific endurance capacity (12, 13). Test results of participants were split into quartiles (very low, low, moderate, high) to categorize performance levels.

### 2.3 Video analysis

**Figure 1:**
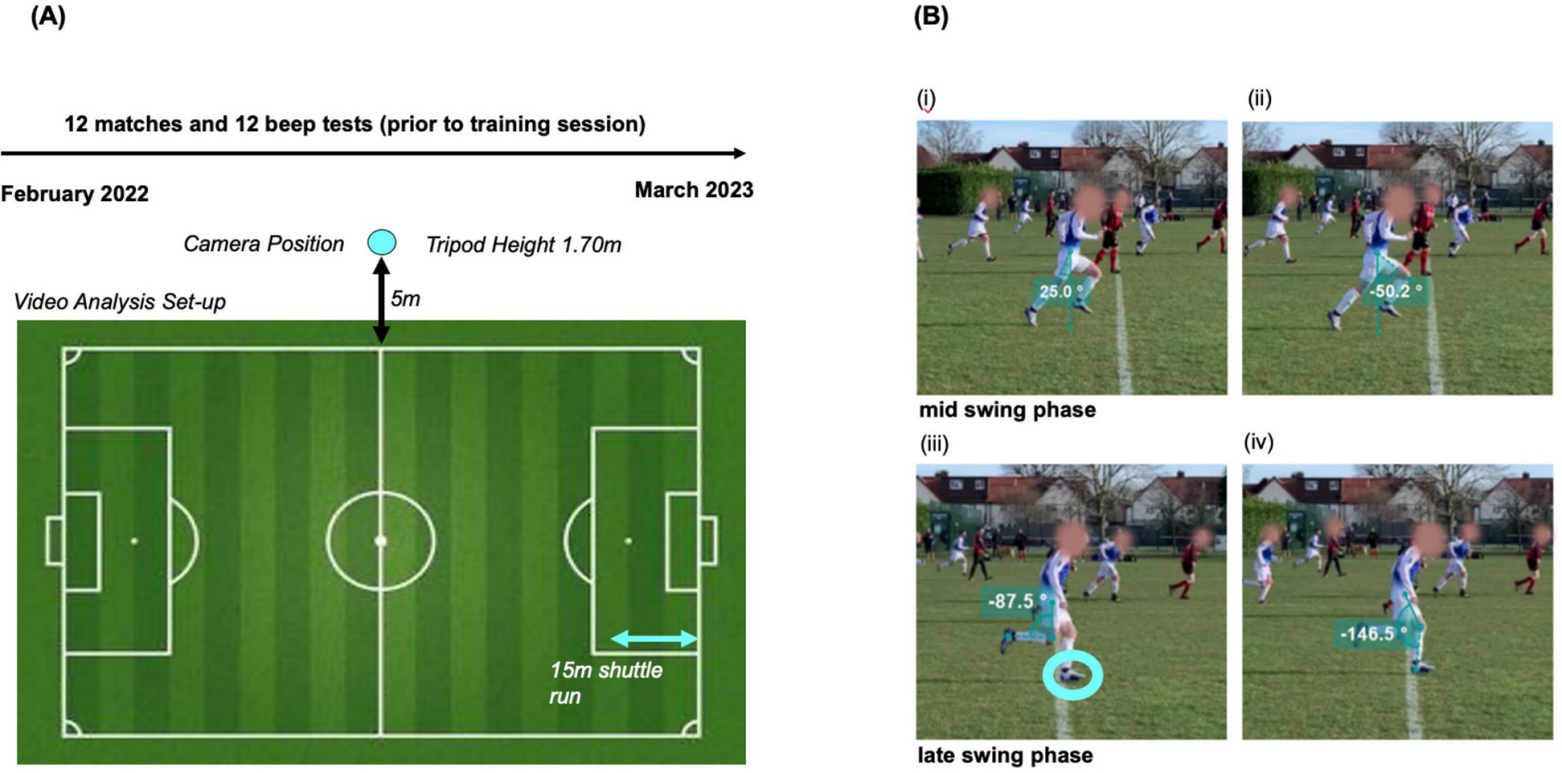
**(A)** Study protocol; general study design, camera positioning, and timeline. The light blue arrow shows the distance of 15 m used for the shuttle run which was done prior to every training session. **(B)** Diagram of angular measurement for kinematic motion analysis. Running: (i) hip extension at its maximal point (here pictured at 25.0°), (ii) hip flexion at its maximal point (here pictured at 50.2°) both in mid swing phase, (iii) knee flexion at its minimum (here pictured at 180–87.5 = 92.5°), and (iv) grounded knee flexion at its maximum (here pictured at 180–146.5 = 33.5°) both in late swing phase. Light blue circle indicates heel strike position.

The use of video recordings of matches allowed for further analysis of football-specific movement characteristics. Hip and knee extension and hip and knee flexion were quantified during sprinting using Kinovea software (14) (Version 2023.1.1, Bordeaux, Nouvelle Aquitaine). All measurements were referenced relative to the anatomical position. Hip angles were assessed from the sagittal viewpoint, measuring the deviation from the body's midline to the midline of the participant's femur. Knee angles were measured at the smallest point between the midlines of the femur and tibia, on both the swinging and the grounded leg during motion (Figure 1). Sprints were defined as a hip extension angle >20° during the mid-to-late swing phase of the running cycle (14, 15). A COD was defined as >90° turns in the transverse plane. Matches were filmed with either an iPhone XS (Apple, Cupertino, CA, USA) or an ORDRO 4K camcorder (Ordro, Shenzhen, China), both positioned on a tripod at midline level and 5 m from the pitch sideline (Figure 1). Video analysis was performed at 25% of real-time video speed. Individual SC including direction (forward, backward, and side-steps) was determined by two independent reviewers by rewinding and fast-forwarding the video footage. When the number of steps differed between reviewers, the median was used. Football-specific movement characteristics were observed over one halftime and extrapolated to individual playing times. The study setup and a diagram illustrating the angular measurement for the kinematic motion analysis are shown in Figure 1.

### 2.4 Data analysis

Exercise intensity was categorized according to heart rate zones (HRZ) based on percent maximum HR (%HR_max_): 1 = 50%–59.9%, 2 = 60%–69.9%, 3 = 70%–79.9%, 4 = 80%–89.9%, and 5 = 90%–100% (16). Subsequently, HRZ and mean HR were compared across match halves, age groups (36–40, 41–45, 46–50, 51–55, and <56 years), years of competitive football experience (15–22, 23–30, 31–38, 39–46, and >46 years), and weekly exercise hours (sedentary 0–2 h, lightly active 3–5 h, moderately active 6–8 h, very active 9–11 h, and extremely active >12 h). The breakdown by age group, years of competitive football experience, and weekly sporting activity was based on five equally sized segments (quintiles) in the respective numerical range. HR data, SC, COD, standing time, number of passes, number of sprints [hip angle during mid-to-late swing phase >20° (Figure 1), measured via Kinovea (14, 15)], and shuttle run test score were analyzed according to the three main field positions (defender, midfielder, striker). For the steps counted by video analysis, an average stride length of 1 m was assumed to estimate the running distance.

### 2.5 Statistics

This study used convenience sampling, a non-probability sampling technique that selects respondents based on their ease of access. Data distribution was confirmed with the Kolmogorov–Smirnov test and reported as mean ± standard deviation (SD) for parametric data or median ± interquartile range (IQR) for non-parametric data. Differences between selected independent variables (match half, age group, years of competitive football experience, and weekly exercise hours) were assessed using MANOVA or Kruskal–Wallis tests. Associations between two variables were calculated using the Pearson correlation coefficient and its non-parametric equivalent, the Spearman rank correlation. A one-way ANOVA—Šídák's multiple-comparisons test—was conducted to assess significance for SC and kilometers traveled between positions. The magnitude of the correlations was based on Evans et al. (17) (<0.20 very weak, 0.20–0.39 weak, 0.40–0.59 moderate, 0.60–0.79 strong, >0.80 very strong). Coefficient of variation (CV) during match was calculated by the following formula: CV = SD/mean × 100.

A significance level of *p* < 0.05 was used for the *α* error. Analyses were conducted using SPSS (version 18.0), and results were presented with GraphPad (version 9.0).

## 3 Results

### 3.1 Demographic data

A total of 91 VF players volunteered to participate in this study. The baseline characteristics are presented in Table 1. The average playing time during the matches was 83 ± 36 min (32–104 min), of which 80/91 (89%) VF players performed ≥60 min.

**Table 1:** Baseline characteristics of 91 veterans football players.

Anthropometric data	Mean ± SD (min–max)
Age (years)	**45** ± 6 (36–62)
Height (cm)	**178** ± 8 (150–196)
Weight (kg)	**84** ± 13 (56–129)
BMI (kg/m^2^)	**26.3** ± 4.0 (20.5–34.8)
Competitive football experience (years)	**32** ± 7 (15–54)
Exercise/week (h)	**5** ± 3 (1–15)
Football training/week (h)	**0.2** ± 0.1 (0–1)
Heart rate	Mean ± SD (min–max)
Maximum HR (bpm)	**183** ± 11 (156–208)
Resting HR (bpm)	**71** ± 12 (40–101)
Mean HR (bpm)	**145** ± 18 (108–177)
Subgroups	Number of subjects (*N* = 91)
Age (years)	
36–40	25
41–45	30
46–50	20
51–55	10
56+	6
Competitive football experience (years)	
15–23	12
24–32	30
33–41	24
42–50	21
51+	4
Exercise*/week (hours)	
0–2, sedentary	7
3–5, lightly active	53
6–8, moderately active	17
9–11, very active	8
12+, extremely active	6
Steps and estimated kilometers covered by field position	Mean ± SD (min–max)
Total	**5,790** **±** **963** (3,232–8,226) steps **5.8** **±** **1.0** (3.2–8.2) km
Forwards (FOR)	**6,384** **±** **1,008** (5,132–7,270) steps
	**6.4** **±** **1.0** (5.1–7.3) km
Midfielders (MID)	**5,953** **±** **1,014** (4,645–8,226) steps
	**6.0** **±** **1.0** (4.6–8.2) km
Defenders (DEF)	**5,388** **±** **1,122** (3,232–7,255) steps **5.4** **±** **1.1** (3.2–7.3) km
Comparison between field positions	Significance (*p*)
FOR vs. DEF, FOR vs. MID, MID vs. DEF	***p*** **=** 0.23*

Heart rate (HR) was measured prior to training, during the 15 m shuttle run test, and during the competitive football match, respectively. Steps were counted and equated to the running distance in kilometers (km) completed in the match. Exercises reported were endurance-based such as walking, running, and cycling. Bold values represent overall means ± SD for the total sample; non-bold values represent subgroup data.

* There was no interaction effect for steps and field position (one-way ANOVA).

### 3.2 Heart rate monitoring

The mean HR during match was 145 ± 18 min^−1^ (108–177 min^−1^) corresponding to 80 ± 8% of HR_max_ (59%–97%). Peak HR during match was 183 ± 11 min^−1^ (156–208 min^−1^) corresponding to 88 ± 6% of HR_max_ (82%–112%). Forwards, midfielders, and defenders spent 68 ± 10%, 58 ± 13%, and 57 ± 14% of match time in HRZ4 and HRZ5, respectively; further percentage of match time spent in HRZs 1, 2, 3, 4, and 5 is presented in Table 2.

**Table 2:** Percentage match time (MT) spent in each heart rate zone (%HR_max_, percentage maximum heart rate) in total and by position, with HRZ4 and HRZ5 representing high-intensity exercise.

Heart rate zone (%HR_max_)	1 (50–59.9)	2 (60–69.9)	3 (70–79.9)	4 (80–89.9)	5 (90–100)
MT total (%)	4 ± 7	12 ± 11	25 ± 11	36 ± 13	21 ± 14
MT 4 and 5 HRZs total (%)				57 ± 14	
MT defenders (%)	4 ± 8	14 ± 12	24 ± 12	37 ± 14	20 ± 14
MT 4 and 5 HRZs DEF (%)				57 ± 14	
MT midfielders (%)	4 ± 8	11 ± 11	25 ± 10	34 ± 10	24 ± 16
MT 4 and 5 HRZs MID (%)				58 ± 13	
MT forwards (%)	2 ± 3	6 ± 5	22 ± 9	45 ± 8	24 ± 11
MT 4 and 5 HRZs For (%)				68 ± 10	

Distribution of HRZ revealed no significant differences between positions. According to the American College of Sports Medicine (ACSM) guidelines, the vast majority (77%) of participants exercised “vigorously” (77%–93% of HR_max_) during the match, while 19% exercised at moderate intensity (64%–76% of HR_max_), and 4% at low intensity (50%–63% of HR_max_; Supplementary File S2) (18). Time spent in the HRZs showed no differences when compared with match half, age group, exercise hours, and years of competitive football experience (Supplementary File S1).

### 3.3 Fitness level

**Figure 2:**
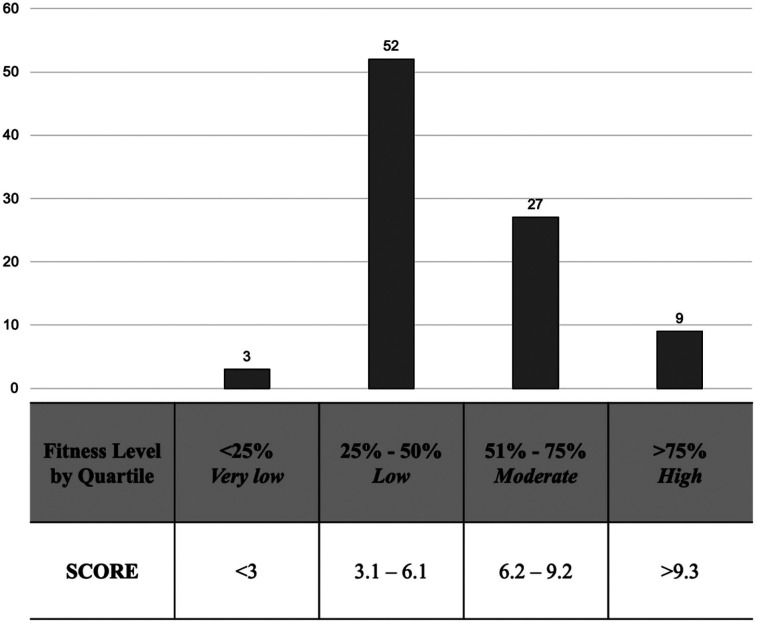
Distribution of the fitness level by quartile of 91 veterans football players.

The players’ average 15 m shuttle run test score was 5.8 ± 2.2, which can be regarded as a low to moderate fitness level (Figure 2). The maximum and minimum scores were 11.5 and 2.4, respectively, with no differences in 15 m shuttle run test scores between positions (FOR 5.9 ± 2.4, MID 6.3 ± 2.6, DEF 6.1 ± 2.2; *p* = 0.91).

### 3.4 Movement characteristics

VF-specific movement characteristics were analyzed in 30/91 (33%) players. These were sampled at random and included players from all separate video files. There were no selected differences in demographic or performance values. Only players with uninterrupted, high-quality video footage were included to ensure data reliability. Overall, the average number of COD was 120 ± 59, the number of passes played was 128 ± 62, and the number of sprints executed was 58 ± 36. The average proportion of standing time per match was 10 ± 2%. Higher SC was positively correlated with an increased number of sprints at moderate strength (*r* = 0.44, *p* = 0.03). Increased time standing correlated to less COD (*r* = −0.22, *p* < 0.001), fewer passes (*r* = −0.27, *p* < 0.001), and fewer sprints made (*r* = −0.39, *p* < 0.001). Comparison of SC with mean HR showed no correlation (*r* < 0.005, *p* = 0.96), while comparison of SC with 15 m shuttle run test scores showed a positive correlation (*r* = 0.38, *p* < 0.009).

### 3.5 Field positions

The positions of VF players were confirmed in 57**/**91 (63%) cases, with 23 defenders, 23 midfielders, and 11 forwards.

**Figure 3:**
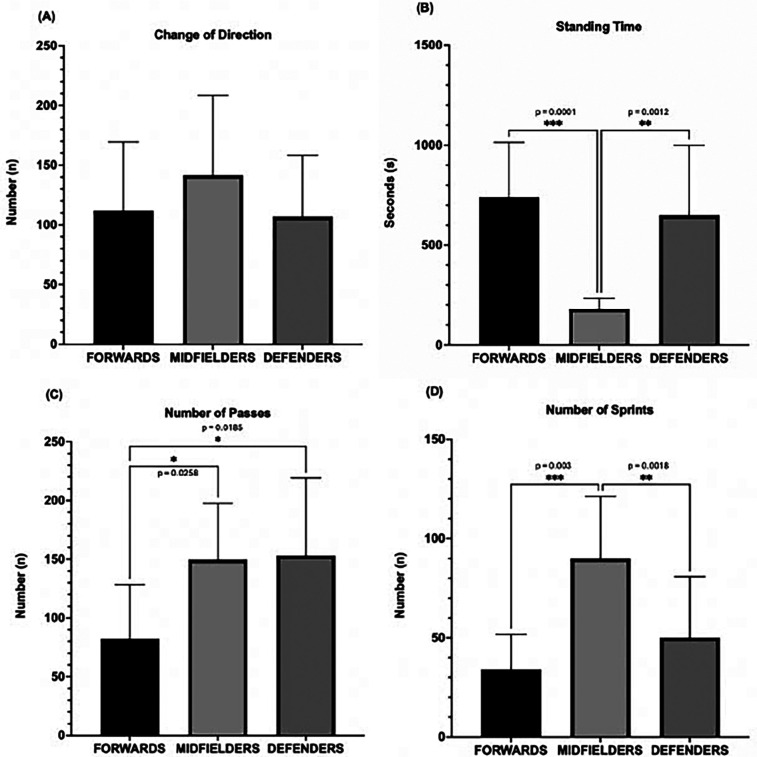
Movement characteristics by position (forwards *n* = 10, midfielders *n* = 10, defenders *n* = 10) **(A)** change of direction, **(B)** standing time (ST), **(C)** number of passes (PS), **(D)** number of sprints (SP) (*p < 0.05, **p < 0.01, ***p < 0.001).

Figure 3 shows no significant differences in COD by position. Forwards and defenders spent more time standing still (740 ± 87 s and 649 ± 111 s, *p* < 0.01) than midfielders (181 ± 17 s, *p* < 0.001), with standing times comprising 14%, 13%, and 3% of match time, respectively. Midfielders and defenders made more passes (150 ± 15 and 153 ± 21) than forwards (82 ± 15, *p* < 0.05). Midfielders also completed more sprints (90 ± 10) than forwards and defenders (34 ± 6 and 50 ± 10, *p* < 0.01).

## 4 Discussion

This study highlights several key findings about VF player activity and match performance. First, the exercise intensity was vigorous for most players (77%), and more than half of the match time was spent in HRZ4 and HRZ5 (≥80% of HR_max_). Second, the estimated match distance covered based on SC was almost 6 km, which correlated moderately with the number of sprints. Third, the average standing time was 10% of match time and significantly higher for forwards and defenders (14% and 13%, respectively) than midfielders (3%). Fourth, midfielders had by far more sprints than defenders and forwards. Fifth, no differences were found between HRZs and match halves, age groups, exercise hours, years of competitive football, or field positions.

### 4.1 Cardiocirculatory strain

The cardiocirculatory strain observed in VF players aligns with previous findings. Wegmann et al. reported similar mean exercise intensity (77% vs. 80% of HR_max_ in this study) and noted that middle-aged VF players have cardiovascular risk profiles comparable to the age-adjusted general population. However, their study observed less match time spent in HRZ4 and HRZ5 (24% and 20%) compared with ours (36% and 22%), possibly due to their players' higher football training volume (1.0 ± 0.6 vs. 0.2 ± 0.1 h per week). A greater training volume may have mitigated the “abrupt overload” effect seen in our subjects, reflected in their relatively low shuttle run scores (5.8 ± 2.2), accurately replicating VO_2max_ levels and serving as a proxy for reduced aerobic capacity and fitness (19). In contrast, Randers et al. (20) reported higher %HR_max_ (82.5%–87.1%) during match time among middle-aged men (mean age 54 years), likely due to their focus on two 60 min 7 vs. 7 training games, while our subjects played 83 min of 11 vs. 11 competitive play. This is to be expected, as different game formats may alter the physical demands of recreational football players (21). Overall, while HR_max_ values are similar across studies of middle-aged men, differences in game format and cardiovascular fitness play a key role in shaping cardiocirculatory responses during competition. It is important to note that the modest variability in cardiovascular fitness across participants likely contributed to differences in HRZ distribution and overall exertional load. While the average exercise intensity was high, less fit individuals may have reached higher HRZs earlier and remained there longer, even at lower running speeds. Conversely, fitter individuals may have tolerated higher workloads with comparatively lower HR responses, thereby diluting potentially larger differences between HR data (6, 22).

### 4.2 Movement characteristics

This study found a positive correlation between steps taken (kilometers completed) and sprints completed. Since previous VF studies did not investigate these parameters, comparisons can only be made to studies on competitive football. Previous research on elite football players has consistently shown that midfielders cover greater distances compared with defenders and forwards and that forwards cover more distance sprinting than all other positions (23, 24). These positional differences seem to be less pronounced in VF players, explicable by the increased fluidity of positioning as an adaptive response to their albeit diminished cardiovascular fitness. In the present study, there were no differences in total running distance between positions. Moreover, we observed similar exercise intensity levels between positions, with 68±10, 58±13, and 57±14 of match time spent in HRZ4 and HRZ5 by forwards, midfielders, and defenders, respectively (Table 2). The findings in elite football seem to portray more distinct differences in physical demands, with forwards performing more high-intensity repetitions (the greatest distance sprinting and speed attained) and shortest pauses between sprints, while defenders and midfielders cover more total distance at lower intensities (23, 25, 26). Our study showed an alternate picture for VF players with midfielders undertaking significantly more sprints (90 per match) than forwards and defenders (34 and 50 sprints per match, respectively). Notably, another study on elite football players found that both forwards and midfielders engage in significantly more sprints than defenders (27). Positional overlap in movement characteristics may also be partly explained by divergent fitness capacities. Players with lower aerobic capacity might avoid sustained high-intensity efforts, resulting in reduced sprint counts, whereas fitter players may compensate for team deficiencies by covering more ground (22). Such interindividual fitness differences, common in middle-aged recreational athletes, can blur positional distinctions typically observed in elite football (23). This variability may also explain the moderate correlation between sprint count and estimated distance covered.

### 4.3 Football and health

Several studies have linked increased daily SC to reduced cardiovascular morbidity and mortality and upheld musculoskeletal function (18, 28). According to the ACSM, the current recommendation for weekly exercise is 150 min of moderate-intensity activity or 75 min of vigorous-intensity activity, which includes walking at approximately 5 km/h (18).

Our findings indicate high-intensity exercise during matches, with 80 ± 8% of HR_max_ and most time spent in HRZ4 and HRZ5. With matches observed lasting 83 min on average, this approximates to the recommended 75 min per week of vigorous activity, fulfilling weekly targets (18, 29).

Despite this, we found no strong correlations between SC and shuttle run score, although when looking at the general population, individuals with higher cardiorespiratory fitness would be expected to take more steps during a match (18, 29). However, compared with daily life activities, football is a game influenced by rules, tactics, playing level, emotions, and game progress. Thus, the fittest players do not necessarily run the most, as shown by a previous study on endurance performance in professional football players, which found no differences between outfield playing positions (30). Moreover, cooperative playing styles among VF players may result in more evenly distributed steps, somehow resembling jogging, which reduces broader fluctuations in HR throughout the match (7), indicating less of an overall cardiocirculatory strain and a lower threat for those at higher risk of a cardiac event (31).

### 4.4 Field position dynamics

To our knowledge, position-specific movement data in VF have not been collected so far, limiting comparisons to age-matched individuals, making only comparisons to studies investigating younger, mostly professional players feasible in this section. Suggestive fluidity of player position was witnessed throughout each half, yet 100% of player positions were verified through the video recording, at initial and post-halftime kickoff. We found no significant positional differences in SC (kilometers completed) among our VF participants. Studies on younger professional players suggest midfielders, key in linking defense and attack, usually cover significantly more distance (32, 33). However, other research indicates elite defenders and forwards cover similar or greater distances than midfielders (25). A previous study focusing on “purposeful movements” of 55 Premier League players found that, similar to our findings, the time standing still was lower in midfielders than forwards and defenders (27). Of note, this study used video recordings with time-motion analysis (27), whereas in the present study, manual joint-angle measuring during running was performed by use of a special software (15, 16), both of which have been proven as reliable methods (34). While in elite football players, no significant differences in the number of passes between forwards and midfielders were observed (32), our study revealed that VF midfielders played substantially more passes (150 ± 15) than forwards (82 ± 15).

The increased activity in VF midfielders (mainly sprinting and therefore the increase in passes) compared with forwards and defenders could be explained by increasing age. Midfielders seem to be less affected by physical decline (35), suggesting that the fittest players in VF are most likely to be considered for the midfield. Furthermore, Andrzejewski et al. found that elite central defenders cover the most distance at low velocities, while external defenders and forwards show similar activity levels to midfielders (moderate to high velocities) (36). Modern tactics might explain this, as defensive lines are more dynamic and involve offensive duties (37).

Unlike elite players with fixed tactical roles, VF players often switch positions during a match due to fatigue, strategy, or to offset less structured play and small squad sizes (6, 22). Nominated roles (e.g., “midfielder” and “defender”) may therefore not reflect actual movement profiles. This fluidity can blur position-specific patterns and complicate comparisons with elite data, where tactical rigidity is greater. Role sharing and spontaneous space coverage may lead to overlapping workloads and technical actions (38), potentially explaining the absence of distance differences and the higher pass count in midfielders, whose central position and adaptability increase involvement. Positional data in VF should thus be interpreted with caution, recognizing roles as functional rather than fixed.

## 5 Methodological considerations

Several limitations are acknowledged. Distance covered was estimated from SC, assuming a uniform stride length of 1 m. This may not reflect true distance, particularly given individual differences in BMI and stride length. However, as SC was primarily used for comparison, a fixed value ensured consistency and reduced complexity. Similar fixed estimations of stride length or speed have been used in other studies for pragmatic distance calculations (24, 39, 40). Future research should validate step-to-distance conversions against gold-standard methods or use GPS for more precise external load measures (41).

In addition, sprints were identified via manual joint-angle analysis using the Kinovea software (14), which, although established, lacks the velocity-based precision that would be achievable with sensor-based systems (28). This may have introduced observer or classification bias.

Only 30 out of 91 players (33%) underwent video-based movement analysis, and we understand that this may have raised the risk of selection bias. However, to reduce the possibility of this, we ensured participants were selected from all videos taken, and we found no demographic or performance differences in the subsample when compared with the total study population. We understand that the overall transferability to younger players is limited.

The study used a convenience sample from a single VF league, limiting generalizability. The exclusively male, field-player cohort restricts applicability to sex-specific differences in cardiovascular responses, musculoskeletal structure, and movement patterns, potentially influencing performance and injury risk findings. Female VF leagues exist in the UK but have far fewer players, with the “Greater London Women's Football League” being the only established organization, making equivalent sampling challenging. Goalkeepers were excluded due to their distinct positional demands, but their omission limits representation, as their activity and physiological profiles differ substantially from field players (11). Including this subgroup in future work could provide a more complete view of VF match demands.

Potential confounders such as medication use, cardiovascular conditions, and prior musculoskeletal injuries were not controlled for. These factors, common in middle-aged populations, can influence HR, exertion, and movement (e.g., beta-blockers blunting HR and cardiac disease reducing performance) (41). Without accounting for them, physiological responses cannot be attributed solely to match demands. Future studies should collect medical histories or stratify analyses accordingly.

While player positions were verified at kick-off, video analysis revealed frequent transitions between roles (e.g., midfield to forward), highlighting the fluid nature of VF play. This positional dynamism, typical of VF, limits direct comparisons with the more rigid role-specific demands seen in elite football. A key strength of this study is its relatively large sample and the multidimensional dataset, which together provide a comprehensive view of the unique physical and kinematic demands in VF and offer a strong basis for future research in ageing athletic populations.

## 6 Conclusions

Middle-aged VF players are exposed to a high cardiocirculatory strain for most of the VF matches. This has certain implications when assessing VF’s health-related value. Certainly, ACSM criteria for vigorous exercise are achieved, giving VF potential preventative efficacy. However, less trained players who may have difficulties pacing themselves properly may exceed their physical limits and potentially put themselves at risk because vigorous exercise is a potential trigger for cardiovascular events (9, 10, 12). One VF match per week might be an option to meet current health-promoting exercise recommendations (19). With regard to field positions, midfielders seem to have the most active part during VF matches. However, fitness level and covered distance did not differ between field positions, which might be indicative of “fluid” positions as a potential unique pattern of VF with a cooperative, inclusive playing style.

## Data Availability

The raw data supporting the conclusions of this article will be made available by the authors, without undue reservation.
