# Supplementary material for: Physical demands and movement characteristics of veterans football players
Source: Front Sports Act Living. 2025 Sep 4;7:1602127. doi: 10.3389/fspor.2025.1602127 (PMC12443858; doi:10.3389/fspor.2025.1602127)
Supplement: Supplementary file 1 [file Datasheet1.pdf]

Supplementary files

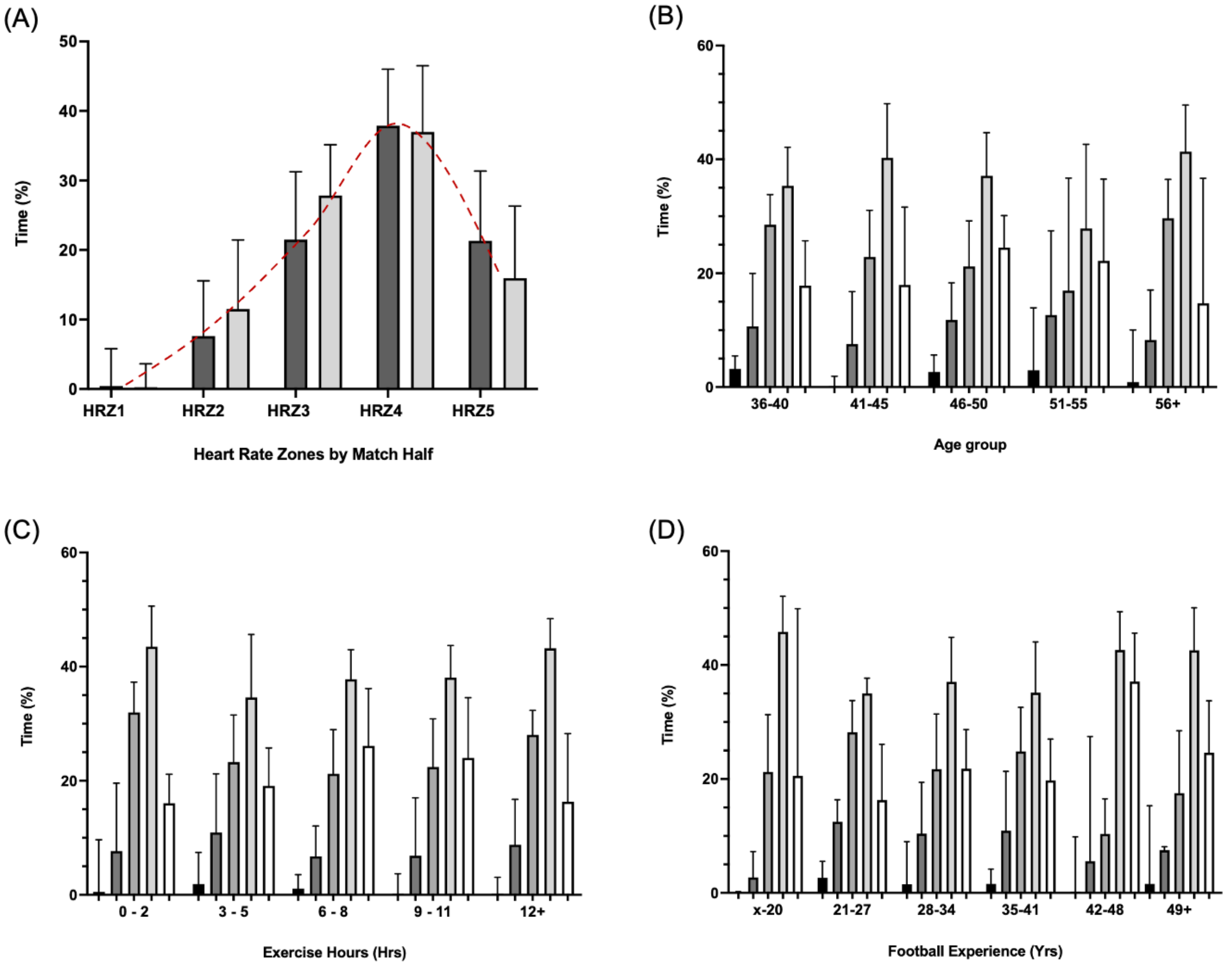

Supplementary file S1: Bar chart of time spent in HRZs 1-5 (n=91); (A) Match Half, (B) Age Group, (C) Exercise Hours, (D) Football Experience.

Players	Exercise Intensity		
	**Light**	**Moderate**	**vigorous**
	HR_{max} 50-63%	***HR_{max} 64-76%***	***HR_{max} 77-93%***
n = 91	2	20	69
	HRR 20-39%	***HRR 40-59%***	***HRR 60-84%***
n = 91	0	41	50

Supplementary file S2: The exercise intensity to which all 91 veterans' football players were exposed was described using percentage maximum heart rate (HRmax) and percentage HRR (heart rate reserve) according to the American College of Sports Medicine (ACSM) criteria[18].
